# Editorial: B Cells in Inflammatory and Neurodegenerative Diseases of the Central Nervous System

**DOI:** 10.3389/fneur.2021.759712

**Published:** 2021-09-13

**Authors:** Francesca Gilli, Roberta Magliozzi, Laura Piccio

**Affiliations:** ^1^Department of Neurology, Dartmouth Hitchcock Medical Center, Geisel School of Medicine at Dartmouth, Lebanon, NH, United States; ^2^Department of Biosciences, Medicine and Movement, University of Verona, Verona, Italy; ^3^Department of Neurology, Washington University in St. Louis, St. Louis, MO, United States

**Keywords:** B cells, neurodegeneration, multiple sclerosis, antibodies, B cell depleting therapies

In recent years, the role of B cells in conditions affecting the central nervous system (CNS) has substantially expanded our perspectives on mechanisms of neuroinflammation and neurodegeneration. The success of B cell-depleting therapies (BCDT) in patients with diseases such as neuromyelitis optica and multiple sclerosis (MS) has underscored the role of B cells in both cellular and humoral-mediated CNS conditions. This Research Topic aimed to provide a comprehensive overview of the functions of B cells in the CNS during homeostasis and in the presence of inflammatory and/or neurodegenerative diseases. Ultimately, the contributions received for this Research Topic covered three main areas (1) antibodies, (2) B cells functions, and (3) therapies, mainly focusing on MS.

Two contributions are focused on the pathogenic role of antibodies. The presence of persistent oligoclonal bands (OCBs) and IgG deposition in demyelinating plaques are essential features of MS pathology. However, their role remains controversial. In their review, Yu et al. hypothesized that circulating IgG1 and IgG3 diffuse across a transiently damaged blood-brain barrier (BBB), contributing to the total intrathecal IgGs and increasing the risk of antibodies-mediated cytotoxicity to CNS cells. However, MS still does not meet the full definition of autoimmune disease, and more studies are needed to clarify the pathological role of antibodies in MS.

Understanding the immunopathogenic functions of antibodies is relevant in a wide range of CNS conditions other than MS. In their original research article, Hang et al. focus on the anti-leucine-rich glioma-inactivated 1 antibody (anti-LGI1) encephalitis, a common autoimmune encephalitis characterized by progressive cognitive impairment. By analyzing the clinical outcome of 21 patients, the authors conclude that considerable antibodies-mediated damage is seen in patients early in the disease, and early and long-term effective immunotherapy can obtain a better cognitive functional prognosis.

Five additional contributions in this Research Topic are focused on the role and function of B cells in CNS conditions. In their review, Chunder et al. discuss the involvement of B cells in two different neuroinflammatory scenarios by drawing parallels between MS and virus-induced neuroinflammation. Both conditions show similar signatures for B cell migration, retention, and regulation in the CNS. Thus, the authors conclude that the basics of B cell biology remain the same independently of the trigger of neuroinflammation, showing a balance between protective and pathogenic functions.

As the nature of the B cell response differs considerably between the stages of the disease, Holloman et al. explore the mechanisms by which B cells contribute to disease progression in primary-progressive MS (PPMS), specifically focusing on cytokine production, antigen presentation, and antibodies synthesis. Authors draw their conclusions based on the analysis of clinical trial data highlighting the existence of a subset of patients with PPMS. The latter have active inflammation contributing to their progressive disability and benefit from BCDTs such as Ocrelizumab. Altogether, the data indicate that BCDTs likely reduce disease progression in PPMS by reducing B cell-mediated inflammation.

Two more review articles focus on the function of specific B cell phenotypes in MS. In their review, Ran et al. unravel the role of regulatory B cells (Breg) and their diversification in demyelinating diseases. An increasing number of studies have confirmed that Breg improve MS. According to the inflammatory microenvironment and the interactions with surrounding cells, Breg can indeed be activated and differentiate into subgroups of cells with beneficial rather than pathogenic functions. Concurrently, DiSano et al. discuss the roles of memory B cells (Bmem), both within the periphery and inside the CNS compartment. Bmem are rising as a critical B cell phenotype in MS due to their antigen experience and rapid response to stimulation. Bmem display diverse effector functions, including antigen presentation to CD4^+^ T cells and precursor to antibody-secreting cells (ASC). On this same line of thought, Negron et al. discuss the evidence supporting the interconnectedness of CD4^+^ T cells, particularly follicular T helper (T_FH_) cells and B cells. T cell-dependent B cell responses originate and take shape in germinal centers (GCs), specialized microenvironments that regulate B cell activation and subsequent differentiation into ASCs or Bmem, a process for which T_FH_ cells are indispensable. GCs represent a critical site of B cell tolerance, and their dysregulation has been implicated in the pathogenesis of several autoimmune diseases, including MS.

Finally, three contributions to this Research Topic focus on different aspects of MS treatment, two specifically discussing BCDTs. Roach and Cross critically review the results of past and ongoing clinical trials of anti-CD20 monoclonal antibodies (mAb) with lytic effects on B cells in MS: Rituximab, Ocrelizumab, and Ofatumumab. The authors review nicely and succinctly also safety profiles, the potential mechanism of action, and alternatives to interfere with B cell function, e.g., anti-CD19 mAb. Complementary to this contribution is the original work by Sempere et al., reporting their real-world experience in a retrospective analysis of 70 MS patients, both relapsing and progressive, treated with Ocrelizumab. Authors report clinical and MRI results, showing that 94% of relapsing patients achieved no evidence of disease activity (NEDA). Despite the overall low number of patients included, this study confirms the effectiveness of BCDTs in the treatment of MS.

The last article on this topic is shifting gear by proposing a new algorithm for progressive multifocal leukoencephalopathy (PML) risk stratification in patients treated with Natalizumab, an anti-α4 integrin mAb interfering with lymphocyte, both T and B cells, migration through the BBB. In this study by Toboso et al., 1,240 people with MS treated with Natalizumab were recruited in 36 European Hospitals to evaluate patients' clinical and demographic characteristics as predictors of PML occurrence. Thirty-five patients developed PML and based on the analysis of B cell-related parameters like anti-lipid specific IgM OCBs and anti-JC virus antibodies, besides disease activity and age, authors established a new algorithm as a PML risk stratification tool for individual patients.

In conclusion, this Research Topic highlights the involvement of B cells in neuroinflammatory diseases. It discusses the evidence supporting B cells' pathogenic immunomodulatory functions in neurological disorders, particularly B cell-directed therapies.

## Author Contributions

FG, RM, and LP wrote this editorial. All authors approved the submitted version of this editorial.

## Conflict of Interest

The authors declare that the research was conducted in the absence of any commercial or financial relationships that could be construed as a potential conflict of interest.

## Publisher's Note

All claims expressed in this article are solely those of the authors and do not necessarily represent those of their affiliated organizations, or those of the publisher, the editors and the reviewers. Any product that may be evaluated in this article, or claim that may be made by its manufacturer, is not guaranteed or endorsed by the publisher.

